# Cladribine Treatment for MS Preserves the Differentiative Capacity of Subsequently Generated Monocytes, Whereas Its Administration *In Vitro* Acutely Influences Monocyte Differentiation but Not Microglial Activation

**DOI:** 10.3389/fimmu.2022.678817

**Published:** 2022-06-06

**Authors:** Tiago Medeiros-Furquim, Sinan Ayoub, Laura J. Johnson, Andrea Aprico, Eze Nwoke, Michele D. Binder, Trevor J. Kilpatrick

**Affiliations:** ^1^ Department of Biomedical Sciences of Cells and Systems, Section Molecular Neurobiology, University Medical Center Groningen, University of Groningen, Groningen, Netherlands; ^2^ The Florey Institute of Neuroscience and Mental Health, University of Melbourne, Parkville, VIC, Australia; ^3^ Department of Neuroscience and Physiology, University of Melbourne, Parkville, VIC, Australia; ^4^ Florey Department of Neuroscience and Mental Health, University of Melbourne, Parkville, VIC, Australia

**Keywords:** multiple sclerosis, cladribine, 2-chlorodeoxyadenosine (2-CdA), innate immunity, neuroinflammation, monocyte, macrophage, microglia

## Abstract

Cladribine (2-chlorodeoxyadenosine, 2CdA) is one of the most effective disease-modifying drugs for multiple sclerosis (MS). Cladribine is a synthetic purine nucleoside analog that induces cell death of lymphocytes and oral cladribine treatment leads to a long-lasting disease stabilization, potentially attributable to immune reconstitution. In addition to its effects on lymphocytes, cladribine has been shown to have immunomodulatory effects on innate immune cells, including dendritic cells and monocytes, which could also contribute to its therapeutic efficacy. However, whether cladribine can modulate human macrophage/microglial activation or monocyte differentiation is currently unknown. The aim of this study was to determine the immunomodulatory effects of cladribine upon monocytes, monocyte-derived macrophages (MDMs) and microglia. We analyzed the phenotype and differentiation of monocytes from MS patients receiving their first course of oral cladribine both before and three weeks after the start of treatment. Flow cytometric analysis of monocytes from MS patients undergoing cladribine treatment revealed that the number and composition of CD14/CD16 monocyte subsets remained unchanged after treatment. Furthermore, after differentiation with M-CSF, such MDMs from treated MS patients showed no difference in gene expression of the inflammatory markers compared to baseline. We further investigated the direct effects of cladribine *in vitro* using human adult primary MDMs and microglia. GM-CSF-derived MDMs were more sensitive to cell death than M-CSF-derived MDMs. In addition, MDMs treated with cladribine showed increased expression of costimulatory molecules CD80 and CD40, as well as expression of anti-inflammatory, pro-trophic genes IL10 and MERTK, depending on the differentiation condition. Cladribine treatment *in vitro* did not modulate the expression of activation markers in human microglia. Our study shows that cladribine treatment *in vitro* affects the differentiation of monocytes into macrophages by modulating the expression of activation markers, which might occur similarly in tissue after their infiltration in the CNS during MS.

## 1 Introduction

Multiple sclerosis (MS) is an immune-mediated demyelinating disease of the central nervous system (CNS) and the leading non-traumatic cause of disability in young adults (1). Current MS therapies can reduce the frequency of relapses mainly by suppressing the immune response (2). Cladribine (2-chlorodeoxyadenosine, 2CdA) is a synthetic purine nucleoside analog that was first designed to treat hematological cancers (3) and is now used to treat several diseases (4), including MS (5). The main effect of oral cladribine administration is lymphocyte depletion (6). However, cladribine treatment leads to drug-free remission in MS patients (7), suggesting the mechanism of action includes potential long-term immune modulation, rather than only cell death.

Cladribine’s impact on the immune system is not limited to lymphocyte depletion, as cladribine also affects innate immune cells. Monocyte-derived dendritic cells are sensitive to death induced by cladribine *in vitro* (8). Moreover, cladribine oral administration leads to a slight reduction in the numbers of circulating NK cells and monocytes in the blood (6). Other than inducing immune cell death, cladribine also exerts immunomodulatory effects in dendritic and T cells (9, 10), and also inhibits cytokine response (11) and migration (12) of mononuclear cells.

Since cladribine crosses the blood-brain barrier (BBB) (13–15), it could also have direct effects on central nervous system (CNS)-resident immune cells (16). As the resident macrophages of the CNS parenchyma, microglia are essential regulators of CNS homeostasis and are implicated in MS (17). Microglia sense their microenvironment and respond with a broad range of activation states, which are thought to play diverse roles in MS pathology (18, 19). On the one hand, scavenger microglia can promote remyelination and tissue repair by phagocytosis of debris and secretion of anti-inflammatory and growth factor molecules (20). On the other hand, inflammatory microglia can secrete pro-inflammatory cytokines and generate reactive oxygen and nitrogen species, which can promote neuroinflammation and neuro-axonal damage (21) and induce neurotoxic reactive astrocytes (22).

Other than the tissue-resident microglia, monocyte-derived macrophages (MDMs) that infiltrate the CNS during inflammation can also contribute to disease progression and appear to play different roles than microglia (23). Studies show that the prevention of monocyte infiltration by CCR2 knockout in mice confers protection against experimental autoimmune encephalomyelitis (EAE) (24–26) and that these infiltrating monocytes do not contribute to the pool of resident microglia (27). Monocytes present distinct phenotypic subsets based on their expression of CD14 and CD16 (28). These subsets show distinct potentials of differentiation (29, 30), and have been suggested to play different roles in MS (26, 31, 32). Moreover, non-classical monocytes (CD14^+^ CD16^++^) have been proposed as novel therapeutic targets in MS (33).

The possibility of drug-free remission in MS patients treated with cladribine suggests a potential long-term immune modulation of microglia and monocytes and their derivatives, rather than simply cell death. However, knowledge on whether cladribine treatment can induce immunomodulatory effects in human microglia and monocytes and their macrophage derivatives is still lacking or limited (34). Therefore, the aim of this study was to determine the immunomodulatory effect of cladribine upon microglia and monocytes and their macrophage-derivatives in the context of MS. Thus, our study had two objectives: to assess the *ex vivo* differentiation potential of monocytes and monocyte-derived macrophages (MDMs) from MS patients treated with cladribine; and to assess how the *in vitro* administration of cladribine influenced the viability and activation profile of primary human adult MDMs and microglia. To this end, we isolated and differentiated monocytes from MS patients before and 19-21 days after treatment with cladribine, as a proxy of how cladribine affects the activation and differentiation of monocytes after their infiltration into the CNS. We also isolated and treated primary human adult MDMs and microglia with cladribine *in vitro* to assess the direct effects of cladribine on the activation of tissue macrophages. Our results suggest that cladribine treatment for MS has limited effects on the subsequent differentiation of circulating monocytes into macrophages. Our data also suggest that during administration, cladribine does not directly activate microglia but can directly affect MDMs differentiation by enhancing the expression of anti- or pro-inflammatory markers, depending on the microenvironment in which they differentiate.

## 2 Material and Methods

### 2.1 Study Design

**Figure 1:**
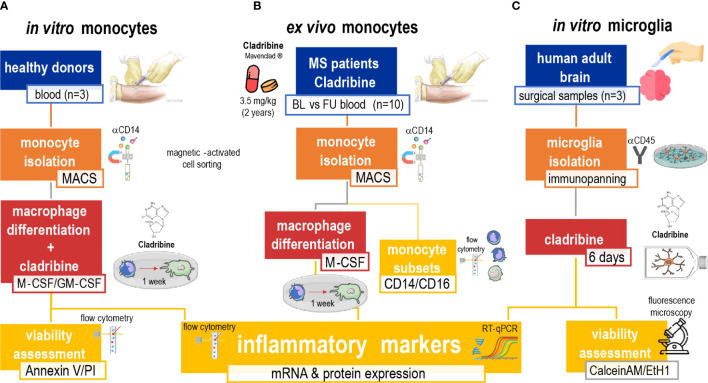
Schematic of the experimental approach. The study was composed of three arms aimed to investigate the effects of cladribine in monocytes, monocyte-derived macrophages (MDMs) and microglia. The first *in vitro* **(A)** approach comprised the isolation of monocytes from healthy donors’ PBMCs by MACS, and M-CSF and/or GM-CSF-induced MDM differentiation. MDMs in differentiation were treated with cladribine for 6 days (total culture period of 7 days). Their viability was analyzed with the annexin V/PI assay by flow cytometry and their activation profile was analyzed by flow cytometry and RT-qPCR. The *ex vivo* **(B)** approach comprised monocytes isolated *via* magnetic-activated cell sorting (MACS) from PBMCs of MS patients undergoing cladribine treatment. These monocytes were analyzed *via* flow cytometry and used to generate M-CSF-induced MDMs *in vitro*, whose activation profile was further analyzed by flow cytometry and RT-qPCR. The second *in vitro* **(C)** approach comprised the isolation of microglia from adult surgical brain samples from patients with temporal lobe epilepsy by immunopanning. Microglia were then treated with cladribine for 6 days. Their viability was assessed by fluorescence microscopy with calcein AM and ethidium homodimer-1 staining, and their activation profile was analyzed by flow cytometry and RT-qPCR. Blue: origin of cells. Orange: isolation technique. Red: *in vitro* culturing and treatment. Yellow: analysis. MS, multiple sclerosis; MACS, magnetic-activated cells sorting; M-CSF, macrophage colony-stimulating factor; GM-CSF, granulocyte-macrophage colony-stimulating factor; RT-qPCR, quantitative reverse transcription-polymerase chain reaction; PI, propidium iodide.

We isolated and analyzed *ex vivo* monocytes and MDMs from MS patients under cladribine treatment, which may indicate how these monocytes behave after infiltration in the CNS during MS pathology. We isolated and treated primary human adult MDMs and microglia with cladribine *in vitro*, to directly assess the effects of cladribine challenge in macrophage differentiation and (microglia) activation. We analyzed the expression of activation markers *via* flow cytometry and RT-qPCR and cell viability *via* flow cytometry and fluorescence microscopy (**Figure 1**).

### 2.2 Cladribine Treatment of MS Patients

To investigate the effects of cladribine treatment, blood samples of diagnosed relapsing-remitting (RR) MS patients receiving oral cladribine (Mavenclad^®^) were collected before (baseline) and 19-21 days after (follow up) starting their first course of cladribine treatment. The recommended cumulative dose of cladribine is 3.5 mg/kg body weight over two years, administered as one treatment course of 1.75 mg/kg per year. The standard treatment course consists of two treatment weeks, one at the beginning of the first month, and one at the beginning of the second month of the respective treatment year. Each treatment week consists of five days on which the patient receives 10 mg or 20 mg as a single daily oral dose, depending on body weight (7). At the time of sample collection, the patients had only taken the first week of the first course of their standard cladribine treatment. Relevant clinical data from patients, including the expanded disability status scale (EDSS) (35), is summarized in **Table 1**.

**Table 1:** Summary of MS patients’ clinical data.

Patient	MS type	Age	Gender	Disease duration	EDSS	Medication during study	Previous immunomodulatory treatments (in years)
#1	RR	38	F	17	0	N/A	β-interferon (12)
#2	RR	50	F	9	0	Coversyl	Fingolimod (7)
#3	RR	54	F	17	4	Oxybutynin	Copaxone (1) and Tysabri (7)
#4	RR	35	F	1	2	N/A	Nil
#5	RR	50	F	13	1.5	N/A	Interferon (3) and Fingolimod (8)
#6	RR	57	M	22	1	N/A	β-interferon (17) and Dimethyl fumarate (4)
#7	RR	47	F	13	1	N/A	β-interferon (12)
#8	RR	45	M	11	2	N/A	β-interferon (11)
#9	RR	46	M	23	3	N/A	β-interferon (3)
#10	RR	50	M	1	2	N/A	Nil

EDSS, expanded disability status scale [ref (35)], RR, relapsing-remitting; N/A, not applicable.

### 2.3 Cell Isolation, Culturing, and Differentiation

#### 2.3.1 Monocyte Isolation

Briefly, blood samples collected in EDTA tubes (BD, #366643) were diluted 1:1 in FACS buffer (Dulbecco’s phosphate-buffered solution without magnesium or calcium (DPBS^-/-^; Gibco, #14190136) containing 2 mM EDTA and 2% heat-inactivated fetal calf serum (HI-FCS; Scientifix life, #FFBS-500)). Peripheral blood mononuclear cells (PBMCs) were obtained by centrifugation [1200 xg, 10 min, room temperature (RT)] using 50 mL SepMate™ tubes (Stemcell technologies, #85460) containing 15 mL Histopaque-1077 (#10771-100, Sigma-Aldrich, St. Louis, MO, USA). PBMCs were then centrifuged (350 xg, 10 min) and washed with FACS buffer. Monocytes were isolated from PBMCs *via* MACS using the QuadroMACS Starting Kit (#130-091-051, Miltenyi Biotec, Macquarie Park, NSW, Australia), which includes CD14 MicroBeads (Miltenyi Biotec, #130-050-201), LS columns (Miltenyi Biotec, #130-042-401) and QuadroMACS™ Separator (Miltenyi Biotec, #130-090-976), according to the manufacturer’s protocol. The purity of MACS-isolated monocytes was determined by the expression of CD14.

#### 2.3.2 Differentiation of Monocyte-Derived Macrophages (MDMs)

To generate MDMs, MACS-isolated monocytes were seeded in 24-well plates at 5x10^5^ cells/well and cultured (at 37°C, 5% CO_2_) for 7 days in RPMI 1640 without L-glutamine (Gibco, #11875085), supplemented with 10% HI-FCS (Scientifix life, FFBS-500), 100 units(μg)/mL penicillin/streptomycin (Gibco, #15140122), MEM-NEAA (Gibco, #11140050), Glutamax (Gibco, #35050061), 100 ng/mL macrophage colony-stimulating factor (M-CSF) (Peprotech, #300-25), and/or 50 ng/mL granulocyte-macrophage colony-stimulating factor (GM-CSF) (Peprotech, #300-03). GM-CSF was used only for experiments with monocytes isolated from healthy donors. Media were not changed for the duration of the experiments.

#### 2.3.3 Microglia Isolation *via* Immunopanning

Primary human adult microglia were isolated for *in vitro* experiments from surgical brain tissue resected for epilepsy treatment. The epileptic focus was not used for the microglia isolation and was separated at the time of resection by the neurosurgeon.

Microglia were isolated *via* immunopanning after tissue enzymatic digestion (as previously described (36, 37), with adaptations). Specifically, 500 mg of brain tissue was placed in a 6-well plate well with 500 μL DPBS^-/-^ (Gibco, #14190136), and minced to ~1 mm^3^ pieces with a scalpel blade. In each well, 10 mL of 95% O_2_ 5% CO_2_-equilibrated papain solution was added. The papain solution consisted of 1x Earle’s balanced salt solution [EBSS; made in house 10x concentrated: 1.16 M NaCl, 54 mM KCl, 10 mM NaH_2_PO_4_•H_2_O, 1% D(+)-glucose and 0.005% phenol red (Sigma-Aldrich-Aldrich P0290)] containing 30% D(+)-glucose (Sigma-Aldrich, #G7021), 1M NaHCO_3_ (Sigma-Aldrich, #S-5761), 50 mM EDTA, 0.2 mg/mL L-cysteine (Sigma-Aldrich, #C7880), 20 U/mL papain (Worthington, #LS03126), and 125 U/mL DNase I (Worthington, #LS002007). A hole was drilled into the plate lid and connected to a CO_2_ gas supply to maintain pH equilibrium. The plate was placed on a 34°C heat block and incubated for 100 minutes with manual shakes every 15 minutes. Digested brains were transferred to 15 mL tubes and, after the tissue had settled, the supernatant was aspirated and replaced by 4.5 mL of low-ovomucoid (low-ovo) solution (1x EBSS containing 30% D(+)-glucose, 1M NaHCO_3_, 1.5 mg/mL BSA (Sigma-Aldrich, #A8806), 1.5 mg/mL trypsin inhibitor (Worthington, LS003086) and 62.5‬ U/mL DNase I). The tissue was allowed to settle again, and the supernatant was replaced by more 4.5 mL low-ovo solution. This step was done four times in total. Four mL of low-ovo solution was added and the tissue chunks were triturated 40 times using a 5 mL serological pipette. The tissue was allowed to settle, and the supernatant removed with a 1 mL pipette. This trituration step was repeated until almost all tissue had been dissociated (3-5 times in total). Dissociated cells were layered on top of 10 ml of high-ovomucoid (high-ovo) solution (1x EBSS containing 30% D(+)-glucose, 1M NaHCO_3_, 5 mg/mL BSA, 5 mg/mL trypsin inhibitor and 25 U/mL DNase I) and centrifuged at 220 xg for 15 min. The cell pellet was then resuspended in 9 ml of DPBS^+/+^ (Gibco, 14040133) containing 0.02% BSA and applied directly to a positive-selection immunopanning dish, previously coated with rat anti-mouse CD45 monoclonal antibodies (30-F11 clone, BD Pharmingen #550539).

To coat the immunopanning dish, a 10 cm sterile petri dish was incubated overnight at 4°C with 10 mL of 50 mM Tris-HCl (pH 9.5) containing 30 μL goat anti-rat IgG (#112-005-167, Jackson ImmunoResearch, Baltimore, PA, USA). The dish was rinsed three times with DPBS^+/+^ (Gibco, #14040133) and incubated with 12 mL of 0.2% BSA containing 20 μL of rat anti-mouse CD45 monoclonal antibodies (clone 30-F11, BD Pharmingen #550539) for 3-5 h at room temperature. The coated dish was rinsed three times with DPBS^+/+^ immediately before incubation with cell suspension.

Cell suspensions (from up to 500 mg of tissue digestion per dish) were allowed to interact with immunopanning dishes coated with rat anti-mouse CD45 monoclonal antibodies (clone 30-F11, BD Pharmingen #550539) for 30 min at room temperature, with gentle shakes every 10 min. Dishes were washed with DPBS^+/+^ five consecutive times to remove unbound cells and debris. Bound cells were trypsinized (10 min at 37°C) with 12 mL DPBS^-/-^ (Gibco, #14190136) containing 500 units/mL trypsin (Sigma-Aldrich, #T9935) [made up in EBSS^-/-/-^ (Sigma-Aldrich, #E7510)]. Because microglia were still strongly adherent, the trypsin solution was discarded, the panning dish was rinsed twice with DPBS^-/-^ and incubated for 1 min on ice with cold microglia culture medium (Dulbecco’s modified eagle medium/nutrient mixture F-12 (DMEM/F12; Gibco, #11330032) supplemented with 10% heat-inactivated FCS (Scientifix life, FFBS-500), 100 units(μg)/mL penicillin/streptomycin (Gibco, #15140122) and 1x minimum essential medium non-essential amino acids (MEM-NEAAs; Gibco, #11140050)), to weaken cell interaction with the dish surface. The microglia were recovered by repeated pipetting and were centrifuged (400g, 10 minutes at RT). Pelleted microglia were resuspended in 0.5 mL of microglia medium supplemented with 40 ng/mL of the CSF1R ligand IL-34 (R&D SYSTEMS, #5265-IL-010), and counted using a hemocytometer. Trypan blue staining (Gibco, #15250061) was used for discrimination of dead cells.

#### 2.3.4 Microglia Culture

For the *in vitro* experiments, immunopanning-isolated microglia were seeded in 96-well plates at 5x10^4^ cells/well, and cultured (at 37°C, 5% CO_2_) for 7 days in DMEM/F12 supplemented with 10% HI-FCS, 100 units(μg)/mL penicillin/streptomycin and 1x MEM-NEAAs in addition to 40 ng/mL IL-34 (R&D SYSTEMS, #5265-IL-010). No media changes were performed for the duration of the experiments. The culture was supplemented with 40 ng/mL IL-34 every second day (days 2, 4 and 6).

### 2.4 *In Vitro* Cladribine Treatment

To assess the effects of cladribine *in vitro*, monocytes isolated on day 1 of differentiation into MDMs, and isolated microglia on day 1 of culture, were treated with cladribine at either 0.01 μM, 0.05 μM or 0.25 μM for 6 days (cultured for 7 days in total). Cladribine active pharmaceutical ingredient (API) (kindly provided by Merck KGaA, Darmstadt, Germany) was reconstituted in DMSO (Sigma-Aldrich, #D2650100) at a concentration of 10 mM, and serially diluted to working concentrations (10 μM or 1 μM) in culture media. The same volumes of DMSO as the highest concentration of cladribine (either 0.05 μM or 0.25 μM) were serially diluted in culture media and added to cells as the vehicle control for the respective experiment.

### 2.5 Flow Cytometry

To determine the purity and immunophenotype of monocytes, the expression of CD14 and CD16 was assessed in PBMCs and MACS-isolated monocytes by flow cytometry. Briefly, cells were resuspended in FACS buffer (DPBS^-/-^ (Gibco, #14190136) containing 2 mM EDTA and 2% HI-FCS and were stained for 15 min at RT with the PE-conjugated monoclonal antibody mouse anti-human CD14 (Miltenyi,130-091-242), and the FITC-conjugated mouse monoclonal anti-human CD16 antibody (Miltenyi, 130-091-244) at a 1:500 and 1:50 final dilution, respectively. For the isotype controls, cells were stained with mouse PE-conjugated IgG2a isotype control (clone S43.10, Miltenyi, 130-113-834) or mouse FITC-conjugated IgM isotype control (clone IS5-20C4, Miltenyi, 130-113-834) at 1:500 and 1:50 final dilution, respectively. Cells were washed once with FACS buffer, centrifuged (350 xg, 5 min) and the pellet was resuspended in FACS buffer for acquisition in a flow cytometer analyzer.

The expression of surface activation markers was determined by flow cytometry in monocyte-derived macrophages at day 7 of culture. Briefly, the MDMs were detached by incubation with pre-warmed (37°C) DPBS^-/-^ containing 5 mM EDTA for 20 min at 37°C, and were recovered by repeated pipetting, pooled with respective supernatant, and centrifuged (350 xg, 5 min). The pellet was resuspended in FACS buffer, and cells were blocked with fragment crystallizable region (Fc) receptor (FcR) human blocking reagent (Miltenyi, #130-059-901) at a 1:5 final dilution, for 10 min at 4-8°C. Blocked cells were stained for 15 min at RT with anti-human CD80 PE-conjugated antibody (clone REA661; Miltenyi, #130-110-270), anti-human CD163 VioBlue-conjugated antibody (clone REA812; Miltenyi #130-112-134) and anti-human Mer APC-conjugated antibody (R&D SYSTEMS, #FAB8912A) at a final dilution of 1:100, 1:50 and 1:11, respectively. Cells were washed once with FACS buffer, centrifuged (350 xg, 5 min), and the pellet resuspended in FACS buffer for acquisition with a flow cytometer analyzer. Dead cells were identified by propidium iodide (PI; Sigma-Aldrich, #P4864-10) (immediately before acquisition, 1 μL of PI solution was added per mL of sample).

To assess microglia enrichment after isolation, the expression of surface markers in immunopanning-isolated microglia was analyzed by flow cytometry. Primary human microglia were immediately detached from immunopanning dishes *via* trypsinization and washed once with FACS buffer. The cells were then centrifuged (350 xg, 5 min) and resuspended in FACS buffer. Cells were blocked for 10 min at 4-8°C with Human TruStain FcX™ (Biolegend, #422302) Fc receptor blocking solution, at a final dilution of 1:20. Cells were subsequently stained with PE-conjugated anti-human CD11b antibody (clone ICFR44, Biolegend, #301306), APC/Cy7-conjugated anti-human CD45 antibody (clone HI30, Biolegend, #304014), APC-conjugated anti-human CD64 antibody (clone 10.1, Biolegend, #305014), and Brilliant Violet™ 421-conjugated anti-human CX3CR1 antibody (clone 2A9-1, Biolegend, #341620) for 15-30 min at 4-8°C, with each antibody at a final dilution of 1:50.

UltraComp eBeads™ (#01-2222-42, Invitrogen, Carlsbad, CA, USA) stained with the conjugated antibodies were used for compensation. All samples were acquired with CytoFLEX S flow cytometer analyzer (Beckman Coulter, Brea, California, United States) and data were processed using FlowJo (version 10.6.1, Becton Dickinson, Ashland, OR).

#### 2.5.1 Annexin V/PI Viability Assay

To assess cell viability *via* flow cytometry, we performed the annexin V/PI assay. Monocyte-derived macrophages at day 7 of culture (day 6 post cladribine treatment) were detached from wells by incubation with DPBS^-/-^ containing 5 mM EDTA and 0.25% trypsin (Gibco, #15090-046) for 5-10 min at 37°C. Trypsin was neutralized by adding culture media (supplemented with FCS) at 4 times the volume of trypsin solution. Cells were harvested by repeated pipetting, combined with supernatant, and centrifuged (350 xg, 5 min, 4°C). The pellet was resuspended in 1x binding buffer, and annexin V and PI staining was performed with the Annexin V-FITC Kit (Miltenyi, 130-092-052), according to the manufacturer’s protocol. All samples were acquired with a CytoFLEX S flow cytometer analyzer (Beckman Coulter), and data were processed using FlowJo V10 software (Treestar).

### 2.6 Immunocytochemistry

Surface marker expression was visualized *via* immunostaining using fluorescence microscopy. Primary human microglia seeded on coverslips in 24-well plates at day 1 of culture were washed once with DPBS^+/+^ and fixed in 4% paraformaldehyde (PFA) for 5 min at RT. Wells were washed three times with MT-PBS (16.3 mM Na_2_HPO•H_2_O, 62.7 mM NaH_2_PO_4_•H_2_O, 148 mM NaCl (Chem-Supply, Brisbane, Queensland, Australia) in MilliQ H_2_O) for 5 min at RT on platform shaker. Next, cells were blocked and permeabilized with blocking buffer [MT-PBS containing 0.3% Triton X-100 and 10% normal goat serum (NGS; Merck Millipore, #S26-100)] for 1 h at RT. Cells were incubated overnight at 4°C with blocking buffer or anti-Iba1 primary antibody (Wako, #019-19741, RRID: AB_839504) diluted 1:1000 in blocking buffer. Coverslips were washed three times with MT-PBS for 5 min on a platform shaker, and incubated with FITC-conjugated goat anti-rabbit secondary antibody (Jackson Immuno, #111-005-144) (1:200 dilution) and Hoechst 33342 nuclear dye (Invitrogen, H3570) (1:1000 dilution) for 1 h at RT on platform shaker in the dark. After staining, coverslips were washed three times with MT-PBS for 5 min on a platform shaker and mounted on microscopy slides using Dako fluorescence mounting medium (Dako, #S3023). Microscopy pictures were acquired with an Axio Imager.M2 (Zeiss) fluorescence microscope, using ApoTome.2 optical sectioning (Zeiss) and an Axiocam 506 mono camera (Zeiss) with ZEN software (Zeiss). Images were processed and analyzed with Fiji software (38).

### 2.7 Calcein AM/Ethidium Homodimer-1 Viability Assay

The viability of primary human microglia at day 7 of culture (day 6 post cladribine treatment) was determined by fluorescence microscopy using the LIVE/DEAD™ Viability/Cytotoxicity Kit (Invitrogen, L3224), according to the manufacturer’s protocol. Briefly, wells containing cells were incubated with 1 mM calcein-AM (Invitrogen, #C3099) and 2 mM ethidium homodimer-1 (EthD-1; Invitrogen, L3224) diluted in DPBS^+/+^ for 30 min at RT in the dark. Microscopy pictures were acquired with an IX81 inverted fluorescence microscope (Olympus) and processed and analyzed with Fiji software.

### 2.8 Gene Expression Analysis (RT-qPCR)

#### 2.8.1 RNA Extraction

Total RNA was extracted from patient MDMs at day 7 of culture (day 6 post cladribine treatment) using the RNeasy Mini Kit (Qiagen, #74104) with on-column DNase digestion (Qiagen, #79254), as per manufacturer’s instructions. Total RNA was extracted from primary human microglia and MDMs from healthy donors at day 7 of culture (day 6 post cladribine treatment) using the RNeasy Plus Micro Kit (Qiagen, #74034). The concentration and purity of the RNA were determined using the NanoDrop 2000 Spectrophotometer (Thermo Fisher Scientific), as per manufacturer’s instructions.

#### 2.8.2 cDNA Synthesis

Total RNA was reverse transcribed to complementary DNA (cDNA) using the TaqMan Reverse Transcription kit (Applied Biosystems, #N8080234), as per manufacturer’s protocol. Reactions of 40 μL were prepared using RNA template (0.5 μg for patients’ MDMs), (0.25 μg for healthy donors’ MDMs), or (0.225 μg for primary human microglia). Random hexamers (at 2.5 μM) were used as primers for the cDNA synthesis. The reaction was incubated in a Peltier thermal cycler (DNA Engine Tetrad 2 Thermal Cycler, Bio-Rad Laboratories) at the following conditions: 25°C for 10 min, 37°C for 30 min, 95°C for 5 min.

#### 2.8.3 Relative Quantification by qPCR

cDNA was amplified using SYBR green PCR master mix (Applied Biosystems, #4309155), as per manufacturer’s protocol. Reactions of 10 μL were prepared using either 12.5 ng (for patients’ samples), or 6.25 ng (for healthy donors’ and microglia samples) of previously generated cDNA (equivalent RNA quantity), and gene-specific primers (Integrated DNA Technologies, see **Table 2**) at final concentrations of 0.8 μM each. Samples were run in triplicates or duplicates, based on sample availability. qPCR was performed using the ViiA 7 Real-Time PCR System (Applied Biosystems) at the following incubation conditions: hold stage 50°C for 2 min, 95°C for 10 min; PCR stage 95°C for 15 sec, 60°C for 1 min, repeat PCR stage for an additional 39 cycles; melt curve stage 95°C for 15 sec, 60°C for 1 min, 95°C for 15 sec.

**Table 2:** List of primer pairs used for qPCR reactions.

Gene	NCBI accession number	Forward primer (5’➔3’)	Reverse primer (5’➔3’)
***18S***	NR_003286.4	CGGCTACCACATCCAAGGAA	GCTGGAATTACCGCGGCT
***IL1B***	NM_000576.2	TACTCACTTAAAGCCCGCCT	ATGTGGGAGCGAATGACAGA
***TNF***	NM_000594.3	AGGACGAACATCCAACCTTC	GTGTCTGAAGGAGGGGGTAA
***IL10***	NM_000572.3	TTAAGGGTTACCTGGGTTGC	TGTCTGGGTCTTGGTTCTCA
***MERTK***	NM_006343.2	ACATCGACCCTGACTCTATAATTGC	TGAACTTCTGCTGTGACCACACT
***CD40***	NG_007279.1	CAGACACCATCTGCACCTGT	AATTGATCTCCTGGGGTTCC

Relative gene expression was determined by the comparative ‘delta CT’ (ΔCT) analysis (39), where an internal control (*18S* rRNA as reference house-keeping gene) was run for each sample for normalization of target gene expression. For data analysis, the ‘delta delta CT’ (ΔΔCT) analysis, i.e. log_2_ fold change (40), was performed using a reference control (patients’ baselines or vehicle).

### 2.9 Statistical Analyses

Statistical analyses were performed using GraphPad Prism 6 (GraphPad Software, Inc.). The statistical tests used for each experiment are detailed in their respective figure. For patient experiments, paired t-tests were performed with *post-hoc* Bonferroni correction for multiple testing where indicated (p^adj^). For *in vitro* experiments with healthy donor’s monocyte-derived macrophages, ordinary two-way ANOVA with either Tukey’s or Sidak’s *post-hoc* test was performed. “n” refers to biological replicate, i.e., each n represents data from a different individual. Only relevant p-values are depicted in figures.

### 2.10 Ethical Statement

Brain tissue and blood samples were obtained with informed consent under the protocol HREC Project 2018.197 (HREC/18/MH/259) Assessment of whether cladribine reconfigures mononuclear cells – approved by the Melbourne Health Human Research Ethics Committee (HREC). The HREC is constituted and operated in accordance with the National Statement on Ethical Conduct in Human Research 2007 (developed jointly by the National Health and Medical Research Council, the Australian Research Council and Universities Australia).

## 3 Results

### 3.1 Effect of Cladribine Treatment in MS Patients

#### 3.1.1 Cladribine Treatment *In Vivo* Does Not Affect Monocyte Numbers or Subsets

**Figure 2:**
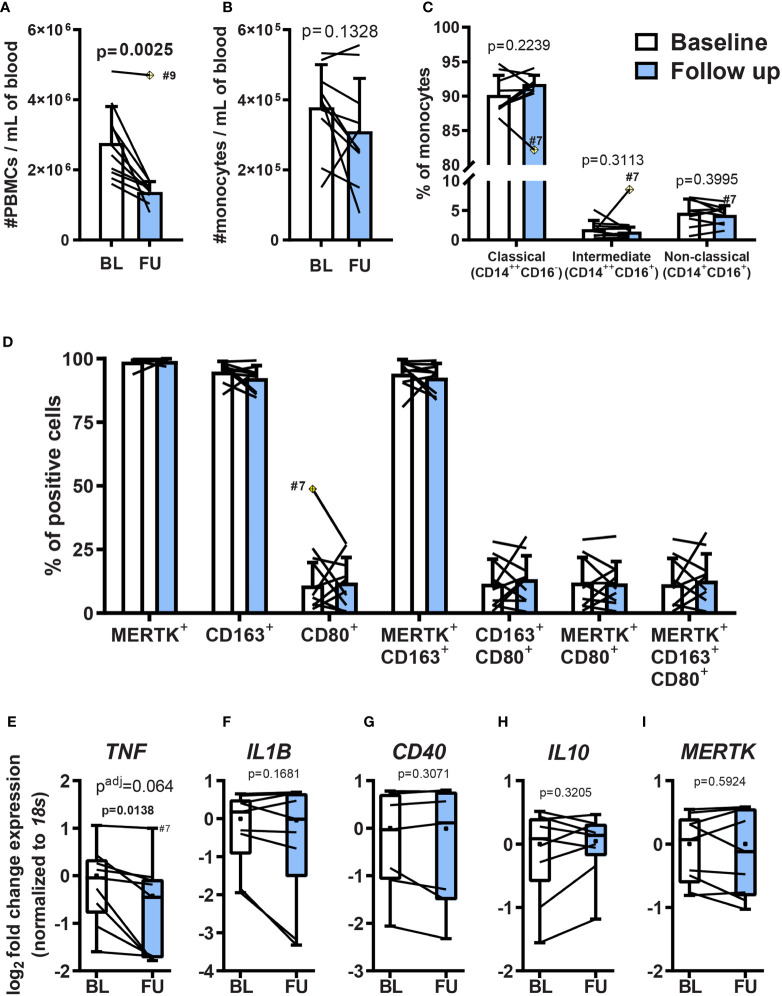
Effects of oral cladribine treatment on circulating PBMCs from MS patients and monocyte *ex vivo* differentiation. Monocytes isolated from MS patients’ PBMCs before (white) and 19-21 days after (blue) the beginning of cladribine treatment were differentiated to MDMs for 7 days with M-CSF. **(A)** The density of peripheral blood mononuclear cells (PBMC) in blood collected from MS patients before (BL; baseline) and 19-21 days after (FU; follow up) the beginning of cladribine treatment. **(B)** The density of MACS-isolated monocytes from freshly isolated PBMCs of MS patients before (BL; baseline) and 19-21 days after (FU; follow up) the beginning of cladribine treatment. **(C)** Monocyte subsets from cladribine-treated MS patients before (white) and 19-21 days after (blue) the beginning of cladribine treatment were determined by CD14 and CD16 surface expression, as analyzed by flow cytometry. **(D)** MDMs of cladribine-treated MS patients were differentiated for 7 days with M-CSF and the expression of pro-inflammatory (CD80) and anti-inflammatory (CD163 and MERTK) activation markers were analyzed by flow cytometry. **(E–I)** Gene expression of activation markers in monocyte-derived macrophages (MDM) from cladribine-treated MS patients. RT-qPCR gene expression analysis of pro-inflammatory **(E–G)** and anti-inflammatory **(H, I)** activation markers. Positive cells were determined according to **(C)** isotype controls and **(D)** FMO controls. **(A–D)** Data depicted as mean (with SD) and lines indicate before-after cladribine treatment, **(A, C)** n=9, **(B–I)** n=10. The yellow crossed diamond shows the statistically significant outlier (ESD, extreme studentized deviate method), which was excluded from the analysis. **(A–D)** Statistical significance was calculated with paired t-test. **(E–I)** Data depicted as median (with quartiles, dot indicates mean), lines indicate before-after cladribine treatment, n=9 (patient #9 was not included due to technical problems). **(E)** n=8, the outlier value #7 follow up (ESD method) was not included in the analysis. **(E–I)** p-values between baseline and follow up were determined by paired t-test and the adjusted p-value (p^adj^) was calculated using the posthoc Holm-Sidak correction for multiple testing.

To determine the influence of Clabribine administered to patients with RRMS on monocytic populations and their derivatives, monocytes were isolated before and after the therapeutic intervention (0.875 mg/kg *per os* (P.O.) distributed over 5 consecutive days) and then studied *ex vivo*. Human monocytes subsets are categorized according to their surface expression of CD14 and CD16, as classical (CD14^++^CD16^-^), intermediate (CD14^++^CD16^+^), and non-classical (CD14^+^CD16^+^). These subpopulations reflect distinct functional roles and differentiation potential (26, 27). To assess the potential influence of cladribine on monocytes and macrophages, monocytes were isolated by magnetic-activated cell sorting (MACS) (**Supplementary Figure 1**) from peripheral blood mononuclear cells (PBMCs) of MS patients before and 19-21 days after the start of cladribine treatment. We analyzed the subsets of these monocytes based on their surface expression of CD14 and CD16 (**Supplementary Figure 2**). Cladribine treatment significantly reduced the concentration of PBMCs in the patients post-cladribine by 1.06x10^6^ [95% confidence interval (C.I.) 0.48x10^6^-1.64x10^6^] cells per ml, representing a 1.72-fold (95% C.I. 1.30-fold to 2.27-fold) reduction (**Figure 2A**). However, cladribine did not significantly alter the concentrations of monocytes (**Figure 2B**) or the proportion between the CD14/CD16 monocytic subsets (**Figure 2C**).

#### 3.1.2 Monocytes Isolated From MS Patients Previously Treated With Cladribine Do Not Exhibit Significantly Altered Differentiative Capacity

Although cladribine treatment did not significantly alter the monocytic population, it could still have influenced their differentiative potential and, therefore, the phenotype of MDMs in the target tissue. To assess this possibility, we differentiated monocytes purified from both the pre and post-cladribine samples *ex vivo* and compared the phenotype of these derivatives. Differentiation was effected using M-CSF, followed by analysis of the surface expression of CD80 as a pro-inflammatory macrophage marker and CD163 together with MERTK as anti-inflammatory markers, quantitated by flow cytometry (**Supplementary Figure 3**). No significant differences in the expression profile of any of these markers were identified amongst the pre and post-cladribine samples (paired t-test, P>0.05) (**Figure 2D**).

To further investigate MDM activation, we analyzed mRNA expression of genes related to pro-inflammatory or anti-inflammatory macrophage activation. We used the gene expression of the pro-inflammatory cytokines *TNF, IL1B*, and the costimulatory molecule *CD40* as markers of pro-inflammatory activation, while the expression of the anti-inflammatory cytokine *IL10*, and the efferocytosis-related receptor *MERTK* were used as markers of anti-inflammatory activation. TNF expression was downregulated 1.5 fold in MDMs derived from MS patients treated with cladribine compared with baseline, although this did not reach significance following correction for multiple testing (p=0.0138, p adj. = 0.064) (**Figure 2E**), while the expression of the other genes remained unchanged (**Figures 2F–I**). These results show that five days of cladribine treatment (+15 days without cladribine, in total 19-21 days) for MS does not modulate the differentiation of monocytes into macrophages with M-CSF.

### 3.2 Cladribine Treatment *In Vitro* Affects the Differentiation of Primary Human Monocyte-Derived Macrophages

**Figure 3:**
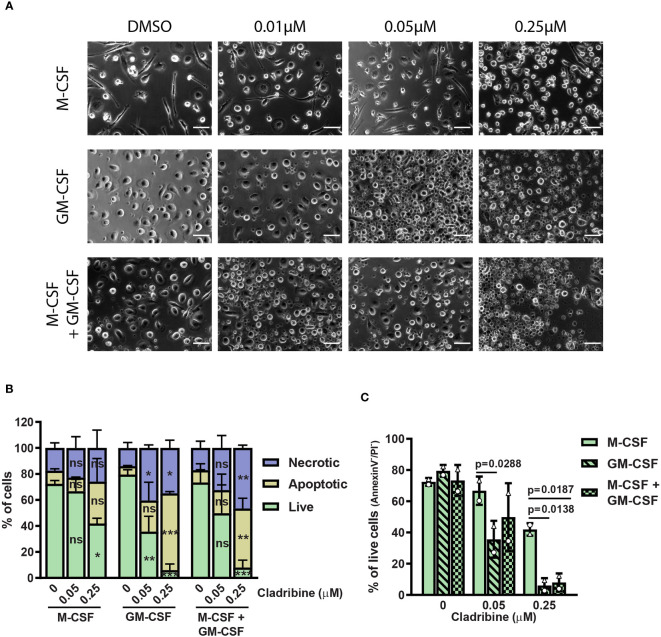
Morphology and viability of monocyte-derived macrophages (MDMs) treated with cladribine *in vitro*. Monocytes isolated from healthy donors were differentiated to MDMs with M-CSF and/or GM-CSF in the presence of cladribine. **(A)** Light microscopy images (phase contrast) of MDMs differentiated with M-CSF and/or GM-CSF with DMSO (vehicle), 0.01µM, 0.05µM or 0.25µM cladribine. Scale bar: 50 μm. **(B)** MDMs viability at day 6 of cladribine treatment *in vitro* was determined by Annexin V/PI staining and analyzed by flow cytometry. Necrotic cells are PI-positive, while apoptotic cells are Annexin V-positive only and live cells **(C)** are negative for both Annexin V and PI. Data depicted as mean (with SD) and symbols in **(C)** (triangle and circle) indicate matched experiments from the same healthy donor’s sample. n=2, **(B)** ns: p > 0.05, *p < 0.05, **p < 0.01, ***p < 0.001, compared to vehicle (DMSO, 0 μM) from the same differentiation condition (CSF group). **(C)** p-values lower than 0.05 between M-CSF and other two differentiation conditions are shown. P-values were determined by ordinary two-way ANOVA with Tukey’s posthoc test.

Cladribine treatment could also exert a direct effect on monocyte differentiation. Therefore, we investigated the effects of cladribine treatment on monocytes *in vitro*, using primary monocytes isolated from healthy donors. We used M-CSF and/or granulocyte-macrophage colony stimulation factor (GM-CSF, also known as colony-stimulating factor 2; CSF2) to generate anti-inflammatory and pro-inflammatory monocyte-derived macrophages, respectively. The M-CSF-differentiated MDMs, but not GM-CSF-differentiated MDMs, developed a spindle-shaped morphology (**Figure 3A**, **Supplementary Figure 4A**), higher surface expression of MERTK and CD163 (**Supplementary Figure 4B**), increased gene expression of *TNF, MERTK*, *IL10*, and reduced gene expression of the costimulatory molecule *CD40* (**Supplementary Figure 4C**). MDMs differentiated with both M-CSF and GM-CSF presented a round and ovoid-shaped morphology (**Figure 3A** and **Supplementary Figure 4A**), and expression profiles similar to GM-CSF differentiated macrophages, as previously described (41), showing reduced expression of MERTK, CD163, and *IL10*, and increased expression of *CD40* (**Supplementary Figures 4B, C**).

To determine the optimal concentration of cladribine for MDM treatment during differentiation *in vitro*, we assessed the influence of two different cladribine concentrations [0.05 μM and 0.25 μM, based on previous studies (8–12) and physiologically-relevant concentrations (13–15)] on cell viability 6 days post-treatment (**Supplementary Figure 5** and **Figures 3B, C**). Cladribine reduced the cell viability in all MDMs, independent of their cytokine exposure, at the concentration of 0.25 μM, while only GM-CSF exposed MDMs had reduced cell viability at the concentration of 0.05 μM (mean difference of 43.90% of live cells, 95% C.I. 16.26% to 71.54%) (**Figures 3B, C**). MDMs differentiated with only M-CSF had a significantly higher proportion of live cells upon 0.05 μM cladribine treatment compared to MDMs differentiated with only GM-CSF (mean difference of 31.15% of live cells, 95% C.I. 3.512% to 58.79%) (**Figures 3B, C**). Upon 0.25 μM cladribine treatment, MDMs differentiated with only M-CSF had a significantly higher proportion of live cells compared to both MDMs differentiated with GM-CSF alone or in combination with M-CSF (respectively, a difference of 35.89% of live cells, 95% C.I. 8.252% to 63.53% and a difference of 33.92% of live cells, 95% C.I. 6.282% to 61.56%) (**Figures 3B, C**).

#### 3.2.1 Cladribine Treatment *In Vitro* Increased the Expression of CD80, CD40, *IL10*, and *MERTK* in Monocyte-Derived Macrophages

**Figure 4:**
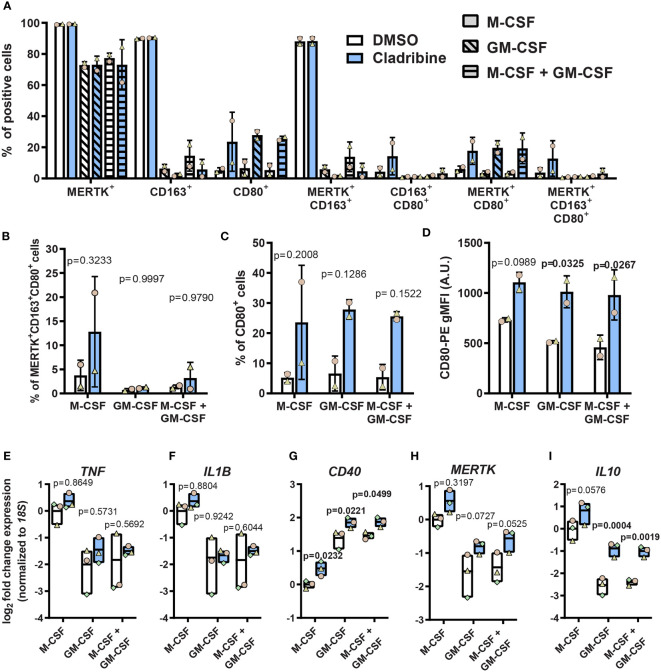
Expression of activation markers in monocyte-derived macrophages (MDMs) treated with cladribine *in vitro*. Monocytes isolated from healthy donors were differentiated to MDMs with M-CSF and/or GM-CSF in the presence of cladribine. **(A–C)** Percentage of MDMs expressing pro-inflammatory (CD80) and anti-inflammatory (CD163 and MERTK) activation markers at day 6 of cladribine treatment *in vitro* as analyzed by flow cytometry. Positive cells were determined according to their fluorescent minus one (FMO) control. **(D)** Levels of CD80 surface protein expression on MDMs treated with cladribine during differentiation *in vitro*, as analyzed by flow cytometry. **(E–I)** RT-qPCR gene expression analysis of **(E–G)** pro-inflammatory and **(H, I)** anti-inflammatory activation markers at day 6 post cladribine treatment *in vitro*. **(A–C)** Positive cells were determined according to FMO controls. **(A–D)** Data depicted as mean (with SD) and symbols indicate matched experiments from the same healthy donor’s sample, n=2. p-values between cladribine 0.05 μM and vehicle (DMSO) were determined by ordinary two-way ANOVA with Sidak’s posthoc test. **(E–I)** Data depicted as mean (with max-min) and symbols indicate matched experiments from the same healthy donor’s sample, n=3. p-values between cladribine 0.05 μM and vehicle (DMSO) were determined by ordinary two-way ANOVA with Sidak’s posthoc test.

To assess the direct effect of cladribine treatment on differentiation and activation of MDMs *in vitro*, we treated monocytes differentiating into MDMs with 0.05 μM cladribine and analyzed the protein expression (**Figures 4A–D**) and gene expression (**Figures 4E–I**) of surface activation markers and cytokines 6 days post-treatment. Cladribine treatment in MDMs differentiated with GM-CSF alone or in combination with M-CSF significantly increased the surface expression of the costimulatory molecule CD80 (**Figure 4D**). Moreover, it significantly induced the expression of the gene encoding the costimulatory molecule *CD40*, regardless of the differentiation factor (fold-change≥1.35 relative to the respective vehicle, p ≤ 0.0499) (**Figure 4G**). On the other hand, cladribine treatment in MDMs differentiated with GM-CSF alone or in combination with M-CSF resulted in significantly increased expression of the anti-inflammatory cytokine *IL10* (fold-change ≥ 2.60 relative to the respective vehicle, p ≤ 0.0019) (**Figure 4I**). There were no significant differences in expression of the other analyzed markers (**Figures 4A, E, F, H**).

In summary, cladribine induced the expression of costimulatory molecules CD80 and *CD40* and the anti-inflammatory cytokine *IL10* in the presence of GM-CSF while inducing only *CD40* expression in M-CSF-differentiated MDMs. These data suggest that cladribine affects macrophage differentiation, depending on the differentiation-inducing factor and, therefore, possibly depending on the microenvironment in which these macrophages are generated.

### 3.3 Effect of Cladribine on Primary Human Microglia *In Vitro*

**Figure 5:**
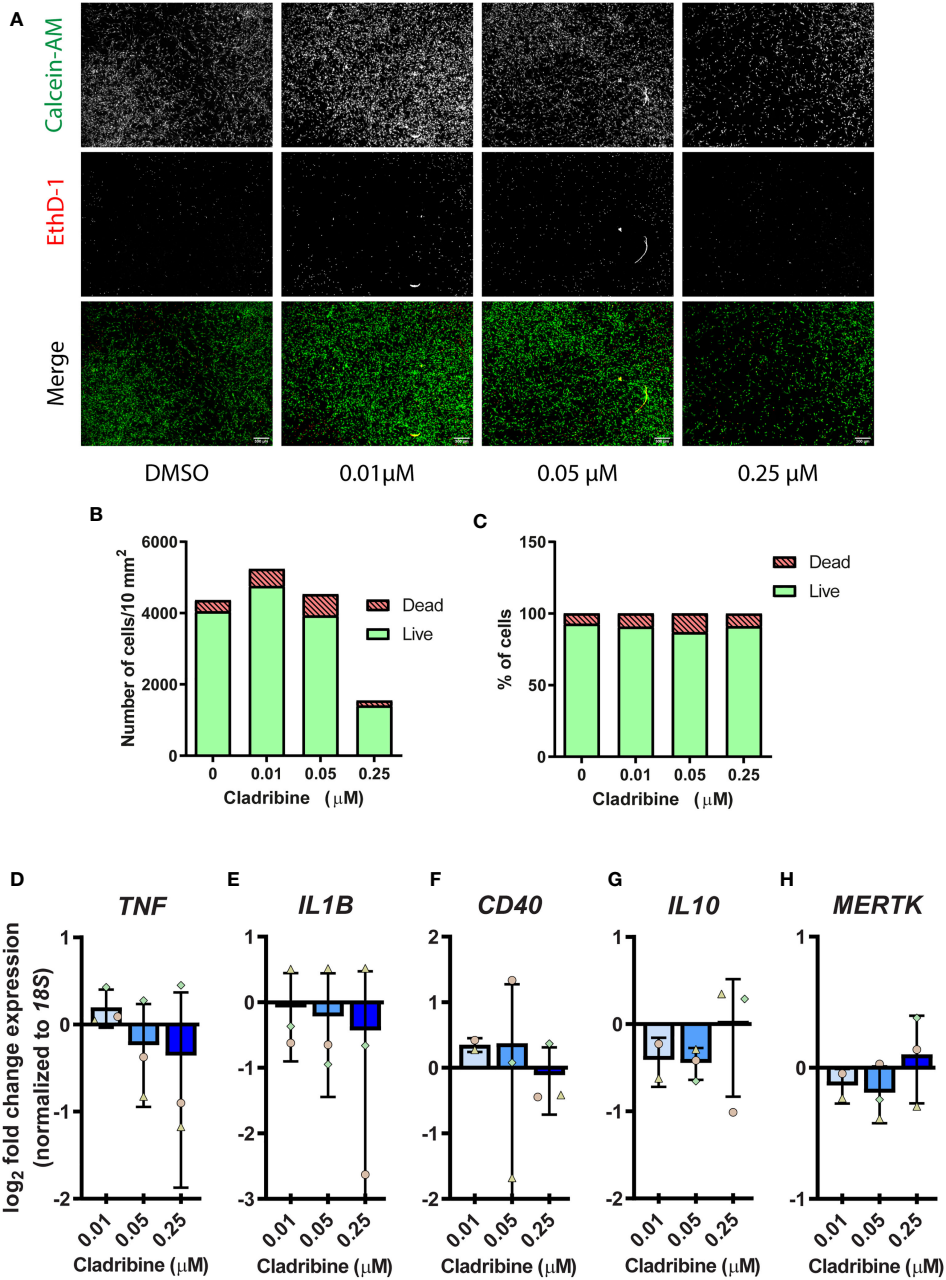
Cell viability and gene expression of activation markers in primary human microglia treated with cladribine. Microglia isolated from temporal lobe surgical resections were treated with cladribine *in vitro* for 6 days. **(A)** Fluorescence microscopy images of human adult microglia stained with calcein-AM (green, live cells) and ethidium homodimer-1 (EthD-1) (red, dead cells) at day 6 post cladribine treatment *in vitro*. **(B, C)** Cell viability of cladribine-treated microglia as assessed by fluorescence microscopy from panel **(A)**, cells positive for calcein-AM are counted as live and positive for EthD-1 are counted as dead. **(B, C)** Data depicted as value, n=1. **(D–H)** RT-qPCR gene expression analysis of **(D–F)** pro-inflammatory and **(G, H)** anti-inflammatory activation markers in microglia at 6 days post cladribine treatment. **(D–H)** Data depicted as mean (+ min/max) and symbols indicate matched experiments from the same brain donor. The fold change expression was calculated for the respective control of each donor. n=3 (except for 0.01 μM for genes *IL10*, *CD40* and *MERTK*, which n=2). Statistical analysis: one-way ANOVA.

Given the aforementioned results suggested a potential role of cladribine in modulating the activation of monocyte-derived macrophages, we argued that cladribine might likely also directly affect the activation of tissue-resident innate immune cells. Therefore, we investigated the effects of cladribine on microglia, from the CNS parenchyma. We isolated CD11b^+^CD45^low^CD64^+^ adult human microglia from surgical brain dissections, obtaining a highly pure and viable (PI^-^) population of cells (**Supplementary Figure 6A**), which were also Iba1^+^ (**Supplementary Figures 6B, C**). To determine the range of cladribine concentration for microglia *in vitro* treatment, we first assessed the effect of three cladribine concentrations (0.01 μM, 0.05 μM, 0.25 μM) on microglial viability by fluorescence microscopy using calcein-AM/ethidium homodimer-1 staining **(** **Figure 5A**). The highest cladribine concentration induced a reduction of 65.23% in the number of live cells as compared to vehicle (DMSO) (**Figure 5B**), but there was no difference in the proprotion between dead and live cells (**Figure 5C**), likely due to the fact that dead cells eventually detach from the plate, as evidenced by the difference in cell density (**Figures 5A, B**). We then assessed the effect of the different cladribine concentrations on microglia activation *in vitro*, based on their gene expression. None of the cladribine concentrations significantly changed the expression of *TNF*, *IL1B*, *CD40*, *IL10*, *MERTK* after 6 days of treatment (**Figures 5D–H**).

## 4 Discussion

Current MS therapies can reduce the frequency of relapses of MS by suppressing the immune response, and the main effect of oral cladribine treatment is lymphocyte depletion. Cladribine treatment leads to drug-free remission (7), and this long-lasting effect cannot be attributed only to lymphocyte depletion. Cladribine has been shown to exert immunomodulatory effects in dendritic and T cells (9, 10), also inhibiting mononuclear cell cytokine response (11) and migration (12), and such effects are thought to contribute to its efficacy in reducing neuroinflammatory relapses. However, the immunomodulatory effects of cladribine treatment on monocyte-derived macrophage differentiation, and whether cladribine can directly modulate the phenotype of human microglia is unknown. Here we have shown that oral cladribine administration to MS patients does not affect monocyte phenotypes or their differentiation to monocyte-derived macrophages (MDMs), corroborating previous data on MDMs differentiation (34). Still, such MDMs showed a moderate downregulation in *TNF* expression compared to baseline. It has been shown that cladribine treatment in combination with lipopolysaccharide (LPS) increases the expression of *TNFR2* in primary neonatal mouse microglia (42), and decreases the secretion of TNF-α in GM-CSF-derived MDMs (34), which suggests that cladribine affects the *TNF* pathway. Nonetheless, our data show that cladribine treatment *in vitro* does not modulate the expression of inflammatory markers, including *TNF*, in primary adult microglia. Moreover, our results also show that cladribine treatment during macrophage differentiation leads to an upregulation of pro-inflammatory or anti-inflammatory activation markers, depending on the differentiation agent (GM-CSF or M-CSF) to which they are exposed. These data suggest that during the administration period and while cladribine is available in tissue, the interaction between cladribine and the CNS microenvironment could determine the inflammatory potential of MDMs that are generated.

Monocytes are short-lived cells derived from hematopoietic precursors and play roles in MS by infiltrating the CNS and differentiating into macrophages (43). In accordance with previous studies (44–46), our results show that cladribine treatment does not alter monocyte total numbers or their subsets (classical, intermediate, and non-classical). Our data shows that monocytes isolated 19-21 days after the start of cladribine treatment generate similar macrophages to the baseline, as evidenced by the comparable expression of the selected surface markers and inflammatory genes. Being short-lived cells, it is reasonable to assume that the analyzed monocytes were most likely generated after treatment and were probably not directly exposed to cladribine. This would imply that any observed effects upon the phenotype of these cells and of their derivatives would downstream to direct effects exerted upon the long-lived progenitor cells in the bone marrow from which they were derived. As shown by previous studies, hematopoietic stem cells can be epigenetically and metabolically reprogrammed after stimulation and retain memory-like features termed innate immune memory (47–49) [reviewed in (50)]. It is possible that similar mechanisms take place during cladribine treatment and lead to the generation of reprogrammed monocytes (51), which would explain the moderate downregulation in basal *TNF* expression in the MDMs. However, challenging such MDMs with inflammatory stimuli such as LPS and cytokines would be necessary to better understand the MDMs inflammatory potential before and after treatment. Further signaling, epigenetic, metabolic analysis, and unbiased approaches covering the whole genome would be necessary to fully determine the potential of cladribine on the reprogramming of monocytes.

The lesions in the MS brain are heterogeneous and dynamic, ranging from pre-active and chronic active to inactive, displaying different levels of neuroinflammation, demyelination and remyelinative potential (52). Mononuclear cells in these lesions also display a range of phenotypes that can contribute to disease pathogenesis or repair. Consistent with this dichotomy, monocyte differentiation can be induced experimentally using either M-CSF or GM-CSF, which are known to generate anti-inflammatory and pro-inflammatory polarized macrophages, respectively (41, 53, 54). For the differentiation of monocytes from MS patients, we used M-CSF because both of the CSF1 receptor ligands, M-CSF and IL-34, are expressed in the brain (55), and CSF1R signaling is necessary for the maintenance of microglia (56). Therefore, using M-CSF to differentiate MDMs would model conditions found in non-inflammatory MS lesions, including those undergoing repair. In contrast, GM-CSF participates in CNS inflammation and autoimmunity (57, 58), and exposure of monocytes and MDMs to this cytokine can be used to model environments in which pathogenesis is active. Therefore, investigating the effects of cladribine on the differentiation of both M-CSF and GM-CSF-derived MDMs could reveal the effects of cladribine on monocytes and their derivatives present in different types of MS lesions. Our data show that cladribine treatment during differentiation induces contrasting outcomes on the expression of activation markers between M-CSF- and GM-CSF-generated macrophages. These results suggest that cladribine effects on monocyte differentiation depend on the microenvironment to which they are exposed. Such heterogeneous effects have implications for MS since neuroinflammation and de/remyelination are dynamic processes occurring throughout disease progression and lesion stages (52).

Macrophages and microglia are plastic and sentinel cells that activate in response to changes in their microenvironment, and their activation phenotype influences their role in disease. Indeed, the adoptive transfer of anti-inflammatory modulated microglia has been shown to be protective in both autoimmune and demyelination mouse models of MS (59), and clearance of debris by microglia is known to be essential for the remyelination process (60). Additionally, microglia-specific knockout of a molecule in the NF-κB inflammatory pathway, and the expansion of neuroprotective microglia *via* CSF1R stimulation, have both been reported to reduce CNS inflammation in the EAE MS model (61, 62). Thus, macrophage/microglial immunosuppressive and phagocytic activities are considered to be neuroprotective (63), while pro-inflammatory and antigen-presenting activation is thought to promote autoimmune inflammation and demyelination.

Two markers of anti-inflammatory and phagocytic activation are MERTK and IL10. The MERTK receptor plays major roles in myelin phagocytosis (64) and efferocytosis (65, 66), and MERTK expression, together with anti-inflammatory activation, which is induced by IL-10, is important for apoptotic cell clearance (66). Our data show that cladribine treatment *in vitro* during GM-CSF-induced MDM differentiation significantly upregulated the expression of the *IL10* but not the *MERTK* gene. However, during differentiation with GM-CSF, cladribine treatment also significantly induced the expression of costimulatory molecules *CD40* and CD80, which are markers of inflammatory activation. In contrast, cladribine treatment during M-CSF-induced differentiation only significantly upregulated the expression of *CD40*, but not of the other tested genes or surface markers. These results indicate that cladribine treatment might influence macrophage activation in different environmental circumstances but in disparate ways, specific to each tissue microenvironment. Interestingly, our data show that GM-CSF-induced MDMs are more susceptible to cell death induced by cladribine compared to M-CSF-derived MDMs, which indicates a possible mechanism that might contribute to cladribine therapeutic efficacy even in a pro-inflammatory environment: selectively reducing the viability of pro-inflammatory MDMs.

A recent study by Mathieson et al. (31) has also investigated the effects of cladribine treatment on MDMs and monocyte-derived dendritic cells generated from healthy volunteers. This study showed that the pre-treatment with 60 nM cladribine significantly reduced the secretion of IL-6 and TNF-α (but not other analyzed cytokines) and also the phagocytic activity of M-CSF-derived MDMs when challenged with LPS. In contrast to our findings, Mathieson et al. identified no significant reduction in cell viability or significant changes in surface expression of, among other markers, CD40 and CD80. Possible reasons for the disparities in the two studies could lie in the: treatment regimens; for the Mathieson study, cladribine was added at day 2 and 5 of differentiation; cladribine was used at 5, 20 and 60 nM, GM-CSF was used at 100 ng/mL, CD40 levels were assessed by cell surface protein expression and cell viability was assessed using a live/dead cell stain, which preferentially stains necrotic but not apoptotic cells. Of particular note, we had identified that cladribine induced significant apoptosis in MDMs (**Figure 4A**), which would be undetected if Annexin V stain had not been used.

Since cladribine can cross the blood-brain barrier (13), cladribine treatment could have direct effects on microglia, the tissue-resident innate immune cells within the CNS parenchyma. Microglial numbers in the healthy CNS are maintained by self-renewal without contribution from cells in the blood; although, as indicated above, monocytes do enter the MS brain to generate MDMs, which exhibit and maintain a phenotype distinct to that exhibited by the resident microglia (23). When microglia die, the adjacent sentinel microglia undergo mitosis, and in this manner, microglial numbers in the CNS are tightly regulated. Previous studies showed that primary rodent microglia are sensitive to cell death by cladribine *in vitro* (67, 68), and another study showed that cladribine treatment does not alter microglial proliferation in the striatum of mice in the EAE model of MS (69). Regardless of whether cladribine treatment affects microglia numbers, it may still affect their activation and, thus, still be of importance for the therapeutic efficacy of cladribine treatment for MS.

In this study, primary adult human microglia did not show any significant modulation of gene expression upon six days of cladribine treatment. However, it is important to note that the high donor variation due to the nature of the brain tissue (from temporal lobe epilepsy patients) can influence these results and will require replication. For instance, the microglia from two out of three donors showed a dose-dependent downregulation of inflammatory genes, which is in line with the *ex vivo* results of modest *TNF* downregulation in patient-derived MDMs. Other studies have also investigated the effect of cladribine on murine microglia activation. One study has shown that cladribine treatment in primary neonatal rat microglia does not change nitric oxide (NO) generation or TNF-α secretion in response to LPS (67). Additionally, another recent study showed that cladribine, in combination with LPS, but not alone, decreases the phagocytic activity and motility of microglia. The same study showed that the higher concentration of 10 μM cladribine for 24h, in combination with LPS, alters the gene expression of inflammatory cytokines (42). However, there was no difference in the protein secretion of the inflammatory cytokines, and the gene expression changes were only observed if in combination with LPS; cladribine did not modulate these inflammatory genes alone or in combination with IL-4. A more recent study showed that cladribine can inhibit cytokine secretion in primary mouse microglia, albeit in high concentrations (10-200 µM) (68). Altogether, our data and these studies suggest that, at physiologically relevant concentrations for the CNS (13–15) cladribine’s immunomodulatory effect on microglia is limited. Moreover, there are significant differences in the these studies: the time of treatment, the drug concentration, and, most importantly, the origin of cells (murine/humans and neonatal/adult). Of note, an ongoing clinical trial (NCT04239820) (70) will help elucidate the clinical implications of cladribine treatment on human microglia of MS patients by TSPO-PET imaging. Finally, the immunomodulatory effects of cladribine upon human microglia might become evident when cells are treated in combination with inflammatory challenges, such as LPS or cytokines, and with analysis of a larger array of microglia activation genes which were not assessed in our study.

Some limitations should be considered in our study. Cladribine treatment *in vitro* most likely does not represent physiological conditions related to apoptotic cell clearance *in vivo* since dead cells are not quickly cleared as in tissue. Therefore, the observation that cladribine upregulates pro- and anti-inflammatory in MDMs *in vitro* might be a result of increased accumulation of dead cells, which induces the expression of pro-phagocytic genes in M-CSF-differentiated MDMs and the expression of costimulatory molecules in GM-CSF-differentiated MDMs. The accumulation of dead cells can be inferred from the flow cytometry data of cladribine-treated GM-CSF-differentiated MDMs, which are not as phagocytic as M-CSF-differentiated MDMs, and showed a higher percentage of apoptotic/necrotic cells at day seven compared to cladribine-treated M-CSF macrophages (**Figure 3B**). Importantly, this limitation is not present in the *ex vivo* differentiation of monocytes from MS patients since the cells were not directly treated with cladribine *in vitro*, and there was no significant loss of cell viability.

Altogether, our results indicate that cladribine treatment for MS has a limited, if any, indirect effect on the differentiation potential of monocytes and a pronounced direct effect on MDMs differentiation but not on the activation of human microglia. More studies contemplating the functionality of such cells are necessary to assess if the alteration in MDMs gene expression leads to an altered phenotype and whether the phenotype of activated human microglia is affected in other ways. In particular, phagocytosis assays could be of great value in assessing these potential effects since macrophage and microglia phagocytotic activity is an important influence upon CNS physiology and disease (71). Indeed, a recent study showed that cladribine reduced the phagocytic activity of GM-CSF-derived MDMs, but only when these cells were challenged with LPS (34). Future studies further elucidating the immunomodulatory effects of cladribine will provide insight into the role of innate immune cells in CNS inflammatory diseases and could potentially indicate further applications of cladribine beyond immune cell depletion.

## Data Availability Statement

The original contributions presented in the study are included in the article/**Supplementary Material**. Further inquiries can be directed to the corresponding author.

## Ethics Statement

The studies involving human participants were reviewed and approved by the Melbourne Health Human Research Ethics Committee (HREC). Written informed consent to participate in this study was provided by the participants’ legal guardian/next of kin.

## Author Contributions

TK conceived the project. TK, MB, TM-F, and SA designed the experiments. SA optimized preliminary protocols, and TM-F carried out the experiments, with support from LJ for flow cytometry and from AA and EN for microglia isolation. TM-F analyzed the data and performed statistical analysis. TM-F, TK, and MB wrote the manuscript. All authors contributed to the article and approved the submitted version.

## Funding

This investigator-sponsored study was funded by Merck Australia (study #MS700568_0039). TK is supported by the Australian National Health and Medical Research Council (NHMRC) as a Leadership Fellow (#APP1175775). SA was supported by Australian Government Research Training Program Scholarship.

## Conflict of Interest

This investigator-sponsored study was funded by Merck Australia, which had the opportunity to comment on the content of the manuscript, but the decision to publish was made independently by the authors.

## Publisher’s Note

All claims expressed in this article are solely those of the authors and do not necessarily represent those of their affiliated organizations, or those of the publisher, the editors and the reviewers. Any product that may be evaluated in this article, or claim that may be made by its manufacturer, is not guaranteed or endorsed by the publisher.
