# Supplementary material for: Cladribine Treatment for MS Preserves the Differentiative Capacity of Subsequently Generated Monocytes, Whereas Its Administration In Vitro Acutely Influences Monocyte Differentiation but Not Microglial Activation
Source: Front Immunol. 2022 Jun 6;13:678817. doi: 10.3389/fimmu.2022.678817 (PMC9207174; doi:10.3389/fimmu.2022.678817)
Supplement: Supplementary file 1 [file DataSheet_1.docx]

# Supplementary Figures


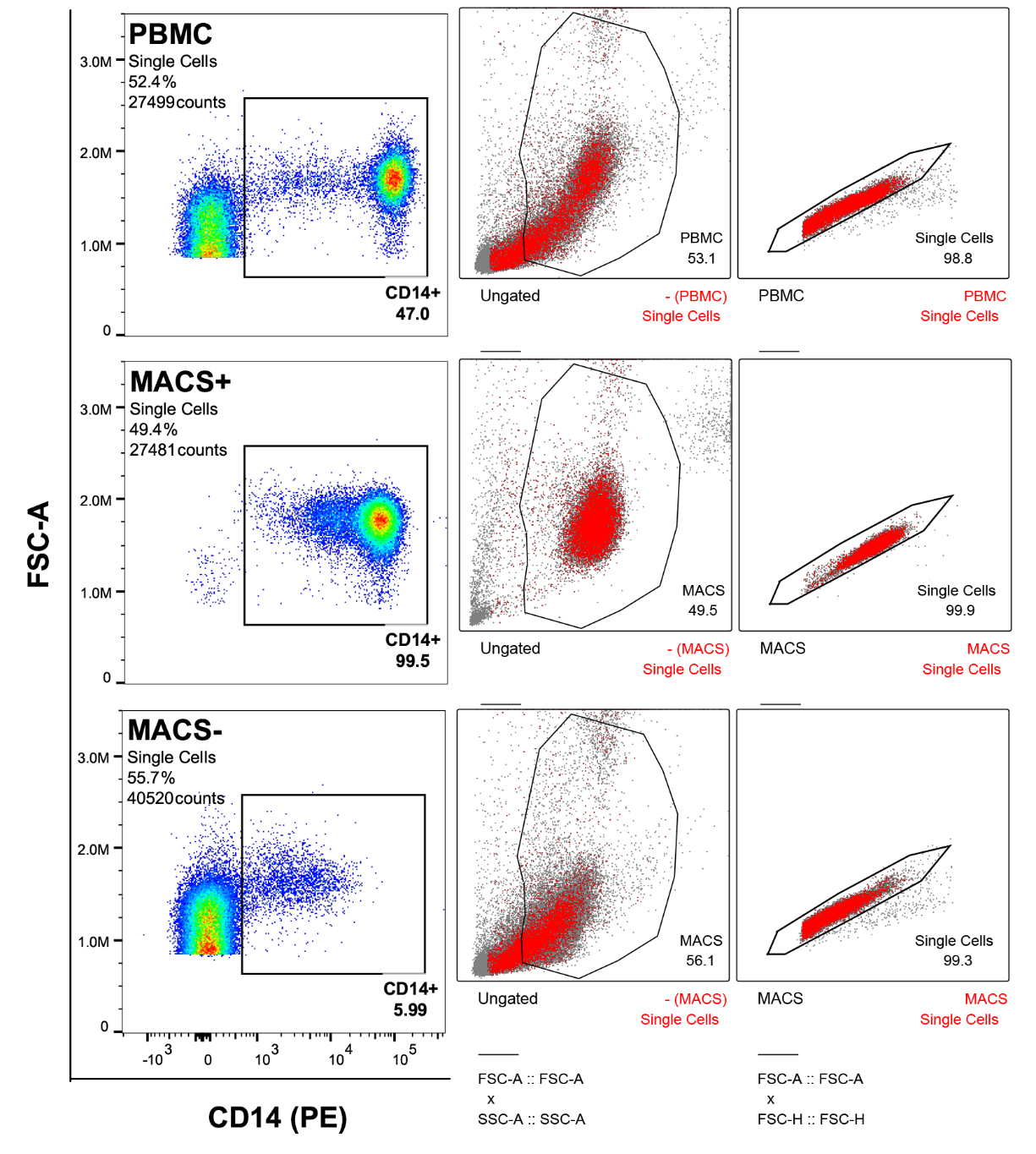


**Supplementary Figure 1.** **Analysis of purity of monocyte isolation from PBMCs via MACS and the density of PBMCs and monocytes in the blood of MS patients**. Monocytes were isolated from PBMCs via MACS on the basis of CD14 positivity. Representative dot plots of flow cytometry analysis of PBMC, cells positively sorted via MACS (MACS+) and negative cells washed during MACS sorting (MACS-) from the blood of MS patients.


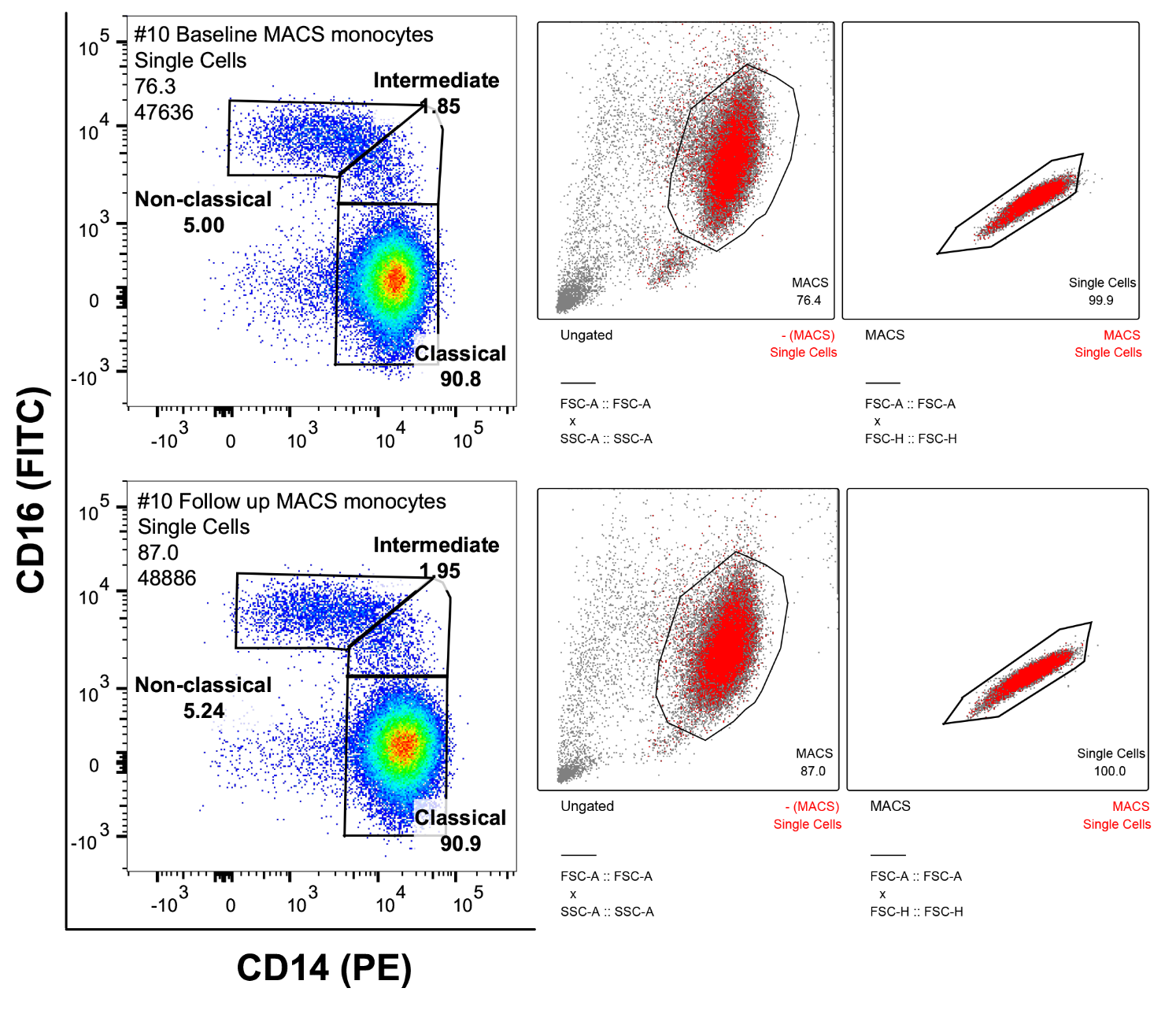


**Supplementary Figure 2.** **Representative dot plot of monocyte subsets, with gating based on CD14 and CD16 surface expression.** Monocytes were isolated via MACS from PBMCs of MS patients receiving cladribine treatment either at baseline or at follow-up between D19-21 post initiation of cladribine and were classified as classical (CD14^++^CD16^-^), intermediate (CD14^++^CD16^+^) and non-classical (CD14^+^CD16^+^) via flow cytometry analysis.


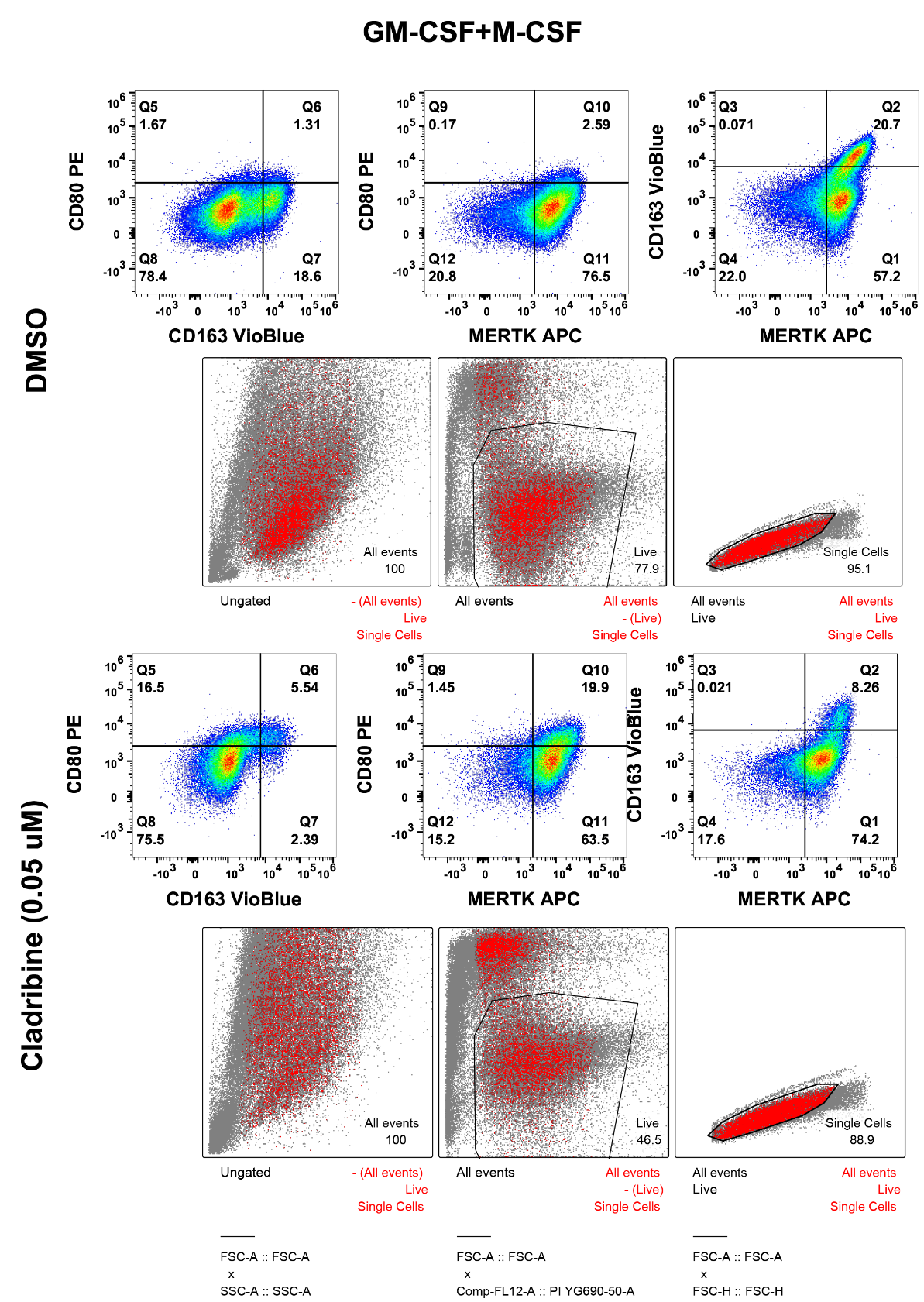


Supplementary Figure 3. Representative dot plots of the surface expression of activation markers in MDMs treated with cladribine during *in vitro* differentiation*.*  MDMs differentiated with M-CSF and GM-CSF were treated with cladribine and the surface expression of CD80, CD163 and MERTK were assessed by flow cytometry. Gating was defined based on fluorescent minus one (FMO) controls.


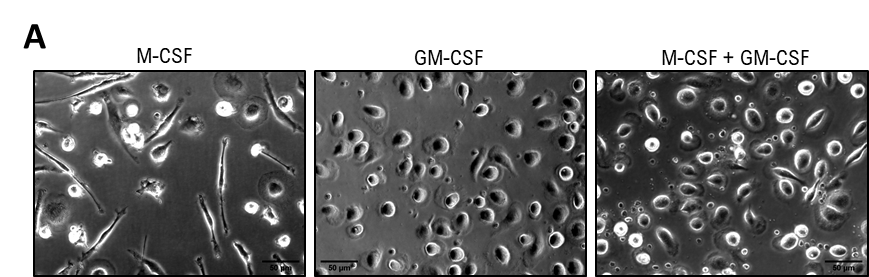

Supplementary Figure 4. The effect of the colony-stimulating factor used to differentiate monocyte-derived macrophages (MDMs) on macrophage activation in the absence of cladribine (A) Light microscopy images of MDMs differentiated with M-CSF and/or GM-CSF. Scale bar: 50 μm. (B) Percentage of cells expressing pro-inflammatory (CD80) and anti-inflammatory (CD163 and MERTK) activation markers at MDMs differentiated with M-CSF and/or GM-CSF, as analyzed by flow cytometry. (C) RT-qPCR gene expression analysis of pro-inflammatory and anti-inflammatory activation markers in MDMs differentiated with M-CSF and/or GM-CSF. Data depicted as (B) mean (with SD) or (C) mean (with max-min). (B) n=2, (C) n=3. p-values between M-CSF and the other two groups were determined by ordinary one-way ANOVA with Dunnett’s posthoc test.


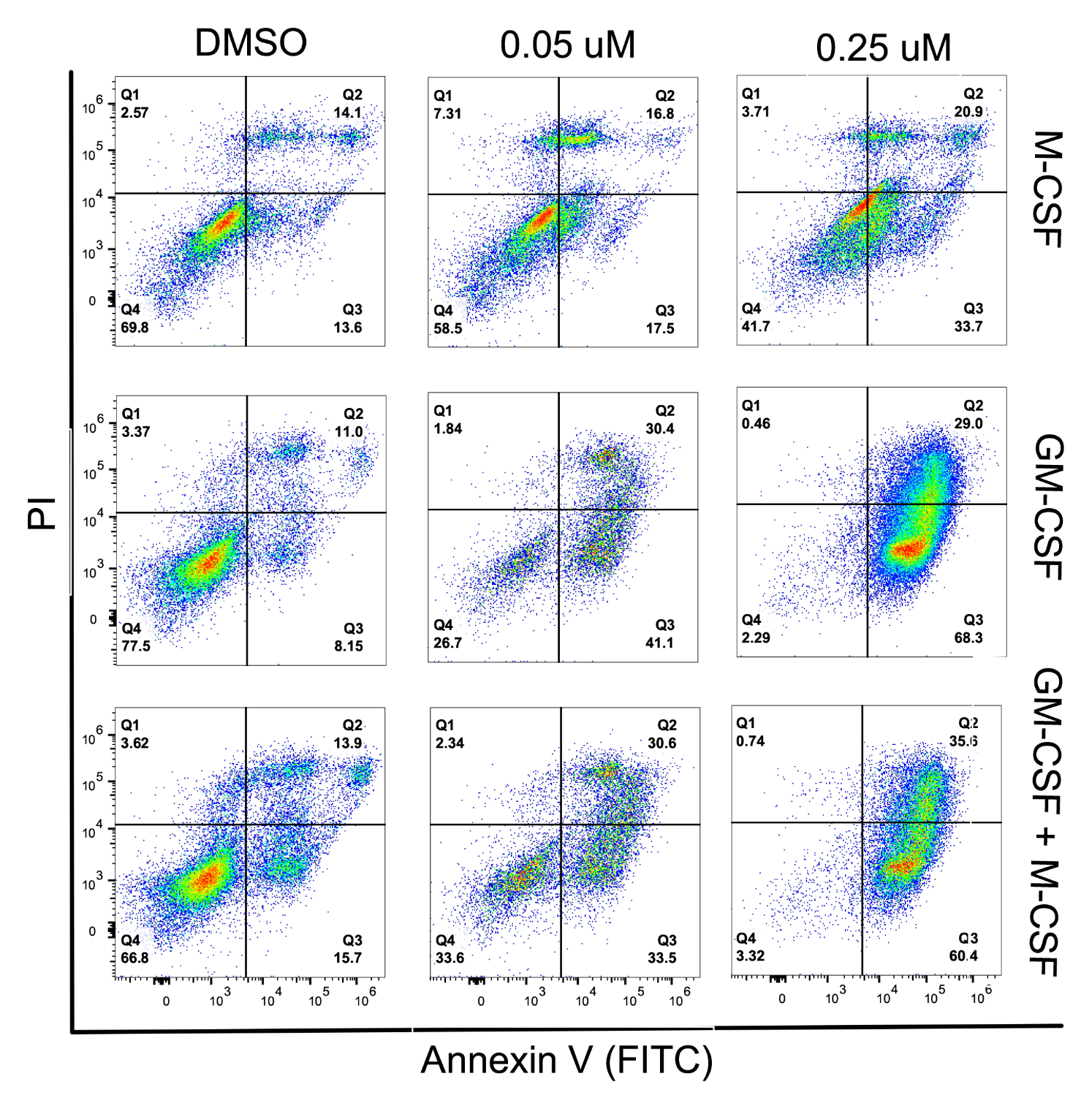


Supplementary Figure 5. Representative dot plot of cell viability analysis via flow cytometry based on annexin V and PI staining. MDMs differentiated with M-CSF and/or GM-CSF were treated with cladribine *in vitro* during differentiation and their viability was assessed at day 6 post-treatment via annexin V/PI assay.


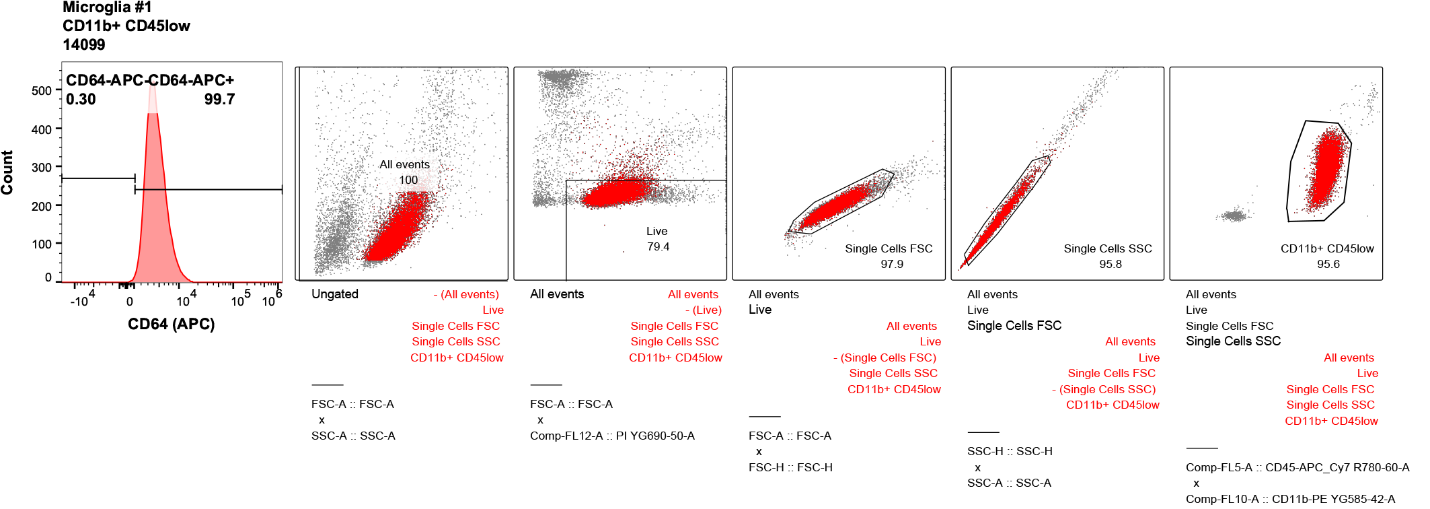


A


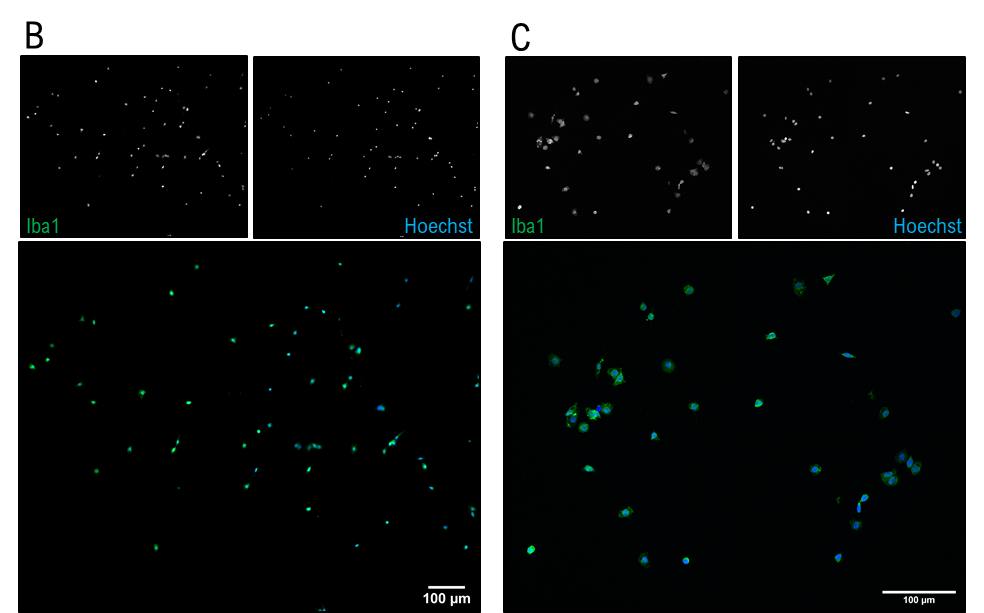


Supplementary Figure 6. Purity and validation of microglia isolation via anti-CD45 immunopanning. Human adult microglia were isolated from surgical brain samples via immunopanning. (A) Gating strategy for flow cytometry analysis of microglia trypsinized from immunopanning plate. PI staining was used for dead cell discrimination. (B, C) Fluorescence microscopy images of immunocytochemistry staining with anti-Iba1 (green) and Hoechst (DNA, blue) of microglia isolated from one human adult brain sample after one day in culture. (B) and (C) show two different magnifications, scale bar: 100 µm.
